# Inverse association of diabetes and dialysis with the severity of femoropopliteal lesions and chronic total occlusion: a cross-sectional study of 2056 cases

**DOI:** 10.1186/s12872-020-01805-6

**Published:** 2020-12-09

**Authors:** Mitsuyoshi Takahara, Yoshimitsu Soga, Masahiko Fujihara, Daizo Kawasaki, Amane Kozuki, Osamu Iida

**Affiliations:** 1 Department of Diabetes Care Medicine, Osaka University Graduate School of Medicine, 2-2 Yamadaoka, Suita City, Osaka 565-0871 Japan; 2 Department of Cardiology, Kokura Memorial Hospital, 3-2-1 Asano, Kokurakita-ku, Kitakyushu City, 802-0001 Japan; 3 Department of Cardiology, Kishiwada Tokushukai Hospital, 4-27-1, Kamoricho, Kishiwada City, Osaka 596-8522 Japan; 4 Cardiovascular Division, Morinomiya Hospital, 2-1-88,Morinomiya, Joto-ku, Osaka City, 536-0025 Japan; 5 Department of Cardiology, Osaka Saiseikai Nakatsu Hospital, 2-10-39, Shibata, Kita-ku, Osaka City, 530-0012 Japan; 6 Cardiovascular Center, Kansai Rosai Hospital, 3-1-69 Inabaso, Amagasaki City, Hyogo 660-8511 Japan

**Keywords:** Peripheral artery disease, Chronic total occlusion, Diabetes mellitus, Dialysis-dependent renal failure

## Abstract

**Background:**

This study aimed to reveal the association of diabetes mellitus and dialysis-dependent renal failure with the lesion severity and chronic total occlusion (CTO) in patients undergoing femoropopliteal endovascular therapy for intermittent claudication.

**Methods:**

This multicenter retrospective study analyzed the data of 2056 consecutive patients with moderate to severe intermittent claudication, who underwent endovascular therapy for de novo lesions in the superficial femoral artery to the proximal popliteal artery between 2010 and 2018 at five cardiovascular centers in Japan. The association of the clinical characteristics with severity of the lesions, as assessed by the Trans-Atlantic Inter-Society Consensus (TASC) II classification, was investigated using the ordinal logistic regression model. Their association with CTO, lesion length, and severity of calcifications was additionally analyzed using the binomial logistic regression model.

**Results:**

The prevalence of diabetes mellitus and dialysis-dependent renal failure was 54.7% and 21.4%, respectively; 12.5% of the patients had lesions corresponding to TASC II class D, and 39.3% of the patients had CTO. Current smoking and severe claudication were associated with more severe lesions assessed according to the TASC II classification; diabetes mellitus and dialysis dependence were inversely associated with disease severity. The adjusted odds ratios of diabetes mellitus and dialysis dependence were 0.82 (95% confidence interval 0.70–0.97; *p* = 0.018) and 0.76 (0.62–0.94; *p* = 0.009), respectively. Diabetes mellitus and dialysis dependence were also inversely associated with CTO (both *p* < 0.05). Furthermore, diabetes mellitus was inversely associated with a long lesion (*p* < 0.05). Diabetes mellitus and dialysis dependence were positively associated with severe calcification (both *p* < 0.05).

**Conclusions:**

Diabetes mellitus and dialysis-dependent renal failure were inversely associated with the lesion severity, as assessed by the TASC II classification, and CTO in patients undergoing femoropopliteal endovascular therapy for intermittent claudication.

## Background

Patients with diabetes mellitus and renal failure have more severe and complex coronary atherosclerotic disease, including higher rates of chronic total occlusion (CTO), compared to those without these comorbidities [1–4]. The presence of CTO is a strong predictor of poor clinical outcomes among patients undergoing percutaneous coronary intervention [1, 5].

The presence of CTO is also a strong predictor of poor clinical outcomes among those undergoing femoropopliteal endovascular therapy for peripheral artery disease (PAD) [6, 7]. However, no clinical studies have examined which comorbidities are associated with complex lesions, especially CTO, in PAD patients. Diabetes mellitus and renal failure are associated with more distally-located (i.e., especially infra-popliteal) arterial disease and more severe calcification [8], but it remains unknown whether the comorbidities are associated with CTO and lesion severity in femoropopliteal segments.

The aim of the current study was to determine the association of diabetes mellitus and dialysis-dependent renal failure with the lesion severity and CTO in patients undergoing femoropopliteal endovascular therapy for intermittent claudication.

## Methods

This multicenter, retrospective study analyzed the data of 2056 consecutive patients between 2010 and 2018 at five cardiovascular centers in Japan, who presented with moderate (Rutherford category 2) to severe (Rutherford category 3) intermittent claudication and underwent endovascular therapy for de novo lesions of the region including the superficial femoral artery to the proximal popliteal artery. The study was conducted in accordance with the Declaration of Helsinki, and was approved by the institutional review boards of the participating institutions. The requirement to obtain any informed consent was waived.

The determination of cardiovascular risk factors was based on the clinical diagnosis according to domestic clinical guidelines. In brief, the presence of hypertension was defined as either (1) having received anti-hypertensive treatment, (2) systolic blood pressure ≥ 140 mmHg, or (3) diastolic blood pressure ≥ 90 mmHg [9]. Hyperlipidemia was defined as either (1) having received anti-hyperlipidemic treatment, (2) fasting triglyceride levels ≥ 150 mg/dl, (3) fasting low-density lipoprotein cholesterol levels ≥ 140 mg/dl, or (4) non-high-density lipoprotein cholesterol levels ≥ 170 mg/dl [10]. Diabetes mellitus was defined as either (1) having received anti-diabetic treatment, (2) fasting plasma glucose levels ≥ 126 mg/dl, (3) casual plasma glucose levels ≥ 200 mg/dl, or (4) hemoglobin A1c levels ≥ 6.5% [11]. Dialysis dependence, i.e., end-stage renal disease on dialysis, included both hemodialysis and peritoneal dialysis. Severity of intermittent claudication was classified into moderate (Rutherford category 2) and severe (Rutherford category 3) [12].

The arterial lesions were evaluated based on angiography before endovascular revascularization. Lesion severity was graded according to the Trans-Atlantic Inter-Society Consensus (TASC) II classification [12]. A long lesion was defined as lesion length ≥ 25 cm [13], and severe calcification was defined as the peripheral arterial calcium scoring system (PACSS) grade 4 [14].

Data are presented as mean ± standard deviations for continuous variables and as percentages for categorical variables unless otherwise indicated. A two-sided *p* value < 0.05 was considered statistically significant. The association of clinical characteristics with the TASC II classification was investigated using the ordinal logistic regression model. We also investigated their association with CTO, long lesions, and severe calcification using the binomial logistic regression model. These associations were presented as odds ratios and 95% confidence intervals (CIs). All statistical analyses were performed using R version 3.6.0 (R Development Core Team, Vienna, Austria).

## Results

**Fig. 1:**
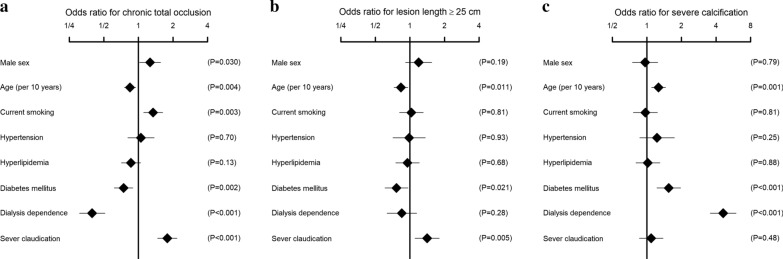
Association of the clinical characteristics with lesion severity. Data are adjusted odds ratios and 95% confidence intervals for chronic total occlusion (**a**), lesion length ≥ 25 cm (**b**), and severe calcification (**c**), derived from the multivariate model in which all the variables listed in the figure were entered as the explanatory variables. Numbers for odds ratios are given in Additional file 1: Table S2

The clinical characteristics of the study population are summarized in Table 1. The prevalence of diabetes mellitus and dialysis-dependent renal failure was 54.7% and 21.4%, respectively; 12.5% of the patients had TASC II class D lesions, and 39.3% of the patients had CTO. The current sample size was calculated to be sufficient to detect an adjusted odds ratio of 1.6 (or its reciprocal 1/1.6 = 0.625) between diabetes mellitus or dialysis dependence and respective lesion characteristics, with a statistical power of more than 80%, under an assumption of the observed prevalence and correlation among covariates (Additional file 1: Table S1). As shown in Table 2, current smoking and severity of claudication were associated with more severe disease as assessed by TASC II classification, whereas diabetes mellitus and dialysis dependence were inversely associated with disease severity. The adjusted odds ratios of diabetes mellitus and dialysis dependence were 0.82 (95% CI 0.70–0.97; *p* = 0.018) and 0.76 (95% CI 0.62–0.94; *p* = 0.009), respectively. No significant interaction effect on the TASC II classification was observed between diabetes mellitus and dialysis dependence (*p* = 0.98). Diabetes mellitus and dialysis dependence were also inversely associated with CTO (Fig. 1a). Furthermore, diabetes mellitus was inversely associated with long lesions (Fig. 1b). By contrast, diabetes mellitus and dialysis dependence were positively associated with severe calcification (Fig. 1c). No significant interaction effect on CTO, long lesions, or severe calcification, was observed between diabetes mellitus and dialysis dependence (*p* = 0.41, 0.33, and 0.14, respectively).

**Table 1:** Clinical characteristics of the study population

*N*	2056
Male sex	1490 (72.5%)
Age (years)	73 ± 9
Current smoker	795 (38.7%)
Hypertension	1769 (86.0%)
Hyperlipidemia	1224 (59.5%)
Diabetes mellitus	1125 (54.7%)
Dialysis dependence	441 (21.4%)
Diabetes mellitus and dialysis dependence	
Diabetes mellitus [−] and dialysis dependence [−]	764 (37.2%)
Diabetes mellitus [−] and dialysis dependence [+]	167 (8.1%)
Diabetes mellitus [+] and dialysis dependence [−]	851 (41.4%)
Diabetes mellitus [+] and dialysis dependence [+]	274 (13.3%)
Severe claudication (Rutherford 3)	1308 (63.6%)
TASC II classification	
Class A	824 (40.1%)
Class B	371 (18.0%)
Class C	603 (29.3%)
Class D	258 (12.5%)
Chronic total occlusion	807 (39.3%)
Lesion length (cm)	14.3 ± 9.9
Lesion length ≥ 25 cm	377 (18.3%)
Severe calcification	419 (20.4%)

Data are presented as mean ± standard deviation or frequency (percentage)

**Table 2:** Association of the clinical characteristics with TASC II classification

	Unadjusted odds ratio	Adjusted odds ratio
Male sex	1.04 [0.87–1.24] (*p* = 0.68)	1.01 [0.85–1.21] (*p* = 0.89)
Age (per 10 years)	0.99 [0.90–1.08] (*p* = 0.75)	0.96 [0.87–1.06] (*p* = 0.38)
Current smoking	1.25 [1.06–1.47] (*p* = 0.007)	1.20 [1.02–1.42] (*p* = 0.032)
Hypertension	0.90 [0.72–1.13] (*p* = 0.35)	0.92 [0.73–1.16] (*p* = 0.48)
Hyperlipidemia	0.95 [0.81–1.12] (*p* = 0.53)	0.92 [0.77–1.08] (*p* = 0.31)
Diabetes mellitus	0.81 [0.70–0.95] (*p* = 0.011)	0.82 [0.70–0.97] (*p* = 0.018)
Dialysis dependence	0.78 [0.64–0.94] (*p* = 0.010)	0.76 [0.62–0.94] (*p* = 0.009)
Severe claudication	1.29 [1.10–1.52] (*p* = 0.002)	1.31 [1.11–1.55] (*p* = 0.001)

Data are presented as odds ratio [95% confidence interval] (*p* value) for TASC II classification. Adjusted odds ratios were derived from the multivariate model in which all the variables listed in the table were entered as the explanatory variables

## Discussion

Femoropopliteal stenosis or occlusion is the most common lesion associated with intermittent claudication [15]. Currently, endovascular therapy is considered as a first-line revascularization strategy for the lesion [6, 13]. The current study demonstrated that diabetes mellitus and dialysis-dependent renal failure were inversely associated with lesion severity, as assessed by the TASC II classification, and the presence of CTO in patients undergoing femoropopliteal endovascular therapy for intermittent claudication; meanwhile the two comorbidities were positively associated with calcification. Diabetes mellitus was also inversely associated with long femoropopliteal lesions. No significant interaction effect on lesion characteristics was observed between diabetes mellitus and dialysis dependence, indicating that the impact of diabetes mellitus and dialysis dependence on respective lesion characteristics was additive.

Diabetes mellitus and renal failure are major risk factors for PAD [8, 16, 17]; a high proportion of patients undergoing femoropopliteal endovascular therapy for intermittent claudication have diabetes mellitus and dialysis-dependent renal failure, as seen in clinical practice [18]. Understanding whether patients with these comorbidities have more severe and complex lesions will help interpretating the clinical outcomes of endovascular therapy.

Diabetes mellitus and dialysis-dependent renal failure were found to be positively associated with severe femoropopliteal calcification. Both comorbidities are major accelerators of calcification in coronary and peripheral arteries [12, 19–21]. Our findings regarding femoropopliteal calcification are in line with this data. In contrast, the association of these comorbidities with CTO seems different between coronary and femoropopliteal arteries. In the coronary arteries, diabetes mellitus and renal failure increase the risk of CTO [1–4], whereas our study demonstrated that these comorbidities had an inverse association with femoropopliteal CTO. Furthermore, diabetes mellitus was inversely associated with long femoropopliteal lesions, which is in contrast to the susceptibility of diffuse coronary lesions in patients with diabetes mellitus [1, 2]. CTO and lesion length are major determinants of lesion severity and complexity. Accordingly, diabetes mellitus and dialysis-dependent renal failure were inversely associated with lesion severity and complexity in femoropopliteal arteries, which was in contrast to the association proved in coronary arteries [1–4].

The pathogenic mechanisms of less severe femoropopliteal lesions in patients with diabetes mellitus and dialysis-dependent renal failure remain unknown. One possible explanation might be the impairment of collateralization. Patients with poor development of collateral vessels might manifest ischemia in the index limb even if occlusive lesions in the main trunk artery are not very severe. Diabetes mellitus is reported to impair the growth of collateral vessels, and various potential mechanisms involving the impairment of arteriogenesis and angiogenesis have been suggested [22]. The contribution of renal failure to impaired collateralization is less clear [23]. Renal failure might have a direct negative effect on collateralization, but also might be a marker of long exposure to uncontrolled diabetes mellitus, since renal failure is a major complication of long-standing diabetes mellitus. Several lines of evidence indicate that, not only impaired vascular flow or perfusion, but also altered skeletal muscle metabolism and inflammatory activation may be responsible for the limb symptoms of PAD [24]. Diabetes mellitus and renal failure might affect these non-vascular mechanistic drivers of claudication [25–28].

Our study had several limitations. First, detailed information about the comorbidities and vessels was limited. No data were available on the etiology of dialysis-dependent renal failure, although we alternatively presented the data on the coexistence of diabetes mellitus and dialysis dependence. Data about the etiology of diabetes mellitus (i.e., type 1 and 2 diabetes mellitus), diabetic neuropathy, and the control of cardiovascular risk factors including diabetes mellitus were also not available. Furthermore, the development of collateral arteries was not assessed since there is no reliable classification system. Second, the current study population was limited to patients with intermittent claudication. It remains unknown whether similar findings were observed in patients with other clinical phenotypes, i.e., asymptomatic patients and those with chronic limb-threatening ischemia. Third, the current study was conducted in Japan. Future studies in other countries are necessary to validate the current findings.

## Conclusions

Diabetes mellitus and dialysis-dependent renal failure were inversely associated with lesion severity, as assessed by the TASC II classification, and CTO in patients undergoing femoropopliteal endovascular therapy for intermittent claudication.

## Supplementary Information

**Additional file 1:** **Table S1.** Statistical powers. **Table S2.** Association of the clinical characteristics with lesion severity.

## Data Availability

The data that support the findings of this study are not publicly available due to ethical reasons but are available from the corresponding author upon reasonable request and with permission of the ethics committee of the participating institutions.

## Abbreviations

CI: Confidence interval
CTO: Chronic total occlusions
PACSS: Peripheral arterial calcium scoring system
PAD: Peripheral artery disease
TASC: Trans-Atlantic Inter-Society Consensus
