# Supplementary material for: Inverse association of diabetes and dialysis with the severity of femoropopliteal lesions and chronic total occlusion: a cross-sectional study of 2056 cases
Source: BMC Cardiovasc Disord. 2020 Dec 9;20:514. doi: 10.1186/s12872-020-01805-6 (PMC7727236; doi:10.1186/s12872-020-01805-6)
Supplement: Supplementary file 1 — Additional file 1: Table S1. Statistical powers. Table S2. Association of the clinical characteristics with lesion severity. [file 12872_2020_1805_MOESM1_ESM.pdf]

Additional file 1: Table S1. Statistical powers

	TASC II classification	Chronic total occlusion	Lesion length ≥25 cm	Severe calcification
Diabetes mellitus	>99.9%	99.9%	97.9%	98.6%
Dialysis dependence	99.4%	97.9%	88.7%	91.0%

Data are statistical powers to detect an adjusted odds ratio of 1.6 (or its reciprocal 1/1.6 = 0.625) between diabetes mellitus or dialysis dependence and lesion severity, under an assumption of the observed prevalence and correlation among covariates in the study population.

Additional file 1: Table S2. Association of the clinical characteristics with lesion severity

	Chronic total occlusion	Lesion length ≥25 cm	Severe calcification
Male sex	1.26 [1.02-1.56] (P=0.030)	1.19 [0.91-1.55] (P=0.19)	0.97 [0.75-1.25] (P=0.79)
Age (per 10 years)	0.85 [0.75-0.95] (P=0.004)	0.84 [0.73-0.96] (P=0.011)	1.26 [1.10-1.45] (P=0.001)
Current smoking	1.34 [1.11-1.63] (P=0.003)	1.03 [0.81-1.30] (P=0.81)	0.97 [0.76-1.24] (P=0.81)
Hypertension	1.05 [0.81-1.37] (P=0.70)	0.98 [0.71-1.36] (P=0.93)	1.23 [0.87-1.74] (P=0.25)
Hyperlipidemia	0.86 [0.71-1.05] (P=0.13)	0.95 [0.75-1.21] (P=0.68)	1.02 [0.80-1.29] (P=0.88)
Diabetes mellitus	0.74 [0.62-0.90] (P=0.002)	0.76 [0.61-0.96] (P=0.021)	1.55 [1.22-1.96] (P<0.001)
Dialysis dependence	0.40 [0.31-0.51] (P<0.001)	0.85 [0.63-1.14] (P=0.28)	4.62 [3.57-5.98] (P<0.001)
Severe claudication	1.79 [1.47-2.17] (P<0.001)	1.41 [1.11-1.80] (P=0.005)	1.09 [0.86-1.38] (P=0.48)

Data are adjusted odds ratios and 95% confidence intervals for lesion severity, derived from the multivariate model in which all the variables listed in the table were entered.
